# Introducing seasonal influenza vaccine in low-income countries: an adverse events following immunization survey in the Lao People's Democratic Republic

**DOI:** 10.1111/irv.12299

**Published:** 2015-01-17

**Authors:** Manilay Phengxay, Sara A Mirza, Rita Reyburn, Anonh Xeuatvongsa, Christian Winter, Hannah Lewis, Sonja J Olsen, Reiko Tsuyuoka, Viengphone Khanthamaly, Francisco S Palomeque, Joseph S Bresee, Ann C Moen, Andrew L Corwin, Lao PDR Field Epidemiology Training Cohort Team

**Affiliations:** a Lao PDR Country Office Western Pacific Regional Office WHOVientiane, Lao People's Democratic Republic; b Influenza Division, U.S. Centers for Disease Control and Prevention (CDC)Atlanta, GA, USA; c National Immunization Program, Lao PDR Ministry of HealthVientiane, Lao People's Democratic Republic; d Lao PDR Country Office, U.S. Centers for Disease Control and PreventionAtlanta, GA, USA

**Keywords:** Adverse events following immunization, influenza vaccine, Lao PDR

## Abstract

**Objective:**

In 2012, Lao PDR introduced seasonal influenza vaccine in pregnant women, persons aged ≥50 years, persons with chronic diseases, and healthcare personnel. We assessed adverse events following immunization (AEFI).

**Methods:**

We used a multistage randomized cluster sample design to interview vaccine recipients.

**Findings:**

Between April and May 2012, 355 902 were vaccinated. Of 2089 persons interviewed, 261 (12·5%) reported one or more AEFI. The most commonly reported AEFIs were local reactions. No hospitalizations or deaths were reported; 16% sought medical care. Acceptance and awareness of vaccination were high.

**Conclusions:**

Following the introduction of seasonal influenza vaccine in Lao PDR, self-reported adverse events were mild.

## Introduction

Influenza vaccines have been used for >60 years and have proven safe and effective.^1^,^2^ However, use of seasonal influenza vaccine in South-East Asia is sparse, with the majority of doses available for cost in the private sector.^3^ In 2012, the World Health Organization's (WHO) Strategic Advisory Group of Experts (SAGE) recommended annual influenza vaccination for five high-risk groups: pregnant women, the elderly, persons with a chronic illness, young children, and healthcare personnel.^4^

The successful deployment of pandemic vaccine in 2009 led the Lao People's Democratic Republic (Lao PDR) Ministry of Health to enter into a public–private partnership with Walgreens Company, facilitated by WHO and U.S. Centers for Disease Control and Prevention (CDC), to distribute 375 000 donated doses of 2011–2012 Northern Hemisphere formulation of the trivalent inactivated seasonal influenza vaccine Fluvirin® (Novartis).^5^,^6^ Lao PDR's National Immunization Program (NIP) has a passive surveillance system to identify adverse events following immunization (AEFI). The Ministry of Health in collaboration with WHO and CDC conducted a survey to actively assess AEFI following introduction of the vaccine.

## Methods

The seasonal influenza vaccine campaign was conducted in Lao PDR from April 23, 2012 to May 8, 2012. Four key populations were targeted: pregnant women, persons aged >50 years, persons with chronic disease, and all healthcare personnel.^6^ Children were not included as a target group because (i) Fluvirin® was indicated for use in persons ≥4 years, and (ii) children aged 4–8 required two doses 4 weeks apart, a logistical challenge deemed not feasible this year. Four provinces were selected for the campaign: Vientiane Capital, Savannakhet, Champasak, and Luang Prabang. Vaccination was voluntary and free to eligible persons. No additional incentive was provided. An information, education, and communication campaign was conducted prior to vaccination.

We estimated statistical power to detect common serious adverse events (e.g., any hospitalization) that might occur as often as 0·5–1·0% and assumed this would not differ by target population.^1^,^7^ Using this frequency, a sample of 2000 vaccinated recipients would allow sufficient precision (relative standard error below 30%) to identify common serious AEFIs. We targeted approximately 2500 vaccine recipients for the survey, estimating 20% non-response. A multistage stratified cluster sampling method was used to obtain a representative sample from the four target populations vaccinated. In stage 1, four districts per province were randomly selected for inclusion. In stage 2, three vaccinated villages with total population >500 persons were randomly selected per district for of 12 villages per province. All pregnant women who received vaccine and at least 15 persons >50 years of age and 15 persons with chronic disease were selected using a random number table from the village vaccine register. Healthcare personnel from three central hospitals were selected in Vientiane Capital province, all provincial hospitals (*n* = 3), and all district hospitals (*n* = 16), and villages in the selected provinces were sampled.

We used a standardized AEFI questionnaire to collect information on demographics, campaign awareness, and AEFI for participants who responded yes to the screening question: “Did you experience any symptoms after seasonal flu vaccination?” An AEFI was defined as having one of the following within 7 days following immunization: soreness, redness or swelling around injection site, fever, headache, sore, red or itchy eyes, nausea, sore throat/hoarseness, rash/hives, fainting, joint pain, itching, general weakness, focal weakness, breathing difficulty, seizure, and paralysis.^8^ Other events were captured in an open-ended question. Multiple symptoms could be reported; frequencies were not mutually exclusive; date of onset and duration were also recorded. Adverse events were categorized as mild (grade 1), moderate (grade 2), severe (grade 3), or life threatening (grade 4) using a modified Solicited Adverse Events scale.^9^ For reporting of rare events, such as anaphylactic shock, stroke, or death, we relied on reporting from the passive NIP AEFI surveillance system. Pregnancy outcome, if available, was assessed. Among those reporting an AEFI, health-seeking behavior and limitation of daily activities were assessed. Lao PDR Field Epidemiology Training (Lao FET) trainees conducted the survey over a three-week period following vaccine administration. Verbal consent was obtained prior to interview.

Descriptive analysis was conducted using EpiData (Odense, Denmark).^10^ Variance and 95% confidence intervals around AEFI point estimates were obtained by the Taylor series linearization method.^7^ The survey was approved by the Lao PDR Ministry of Health and exempted from IRB review as a public health evaluation program.

## Results

A total of 355 902 persons received seasonal influenza vaccine during the campaign; 29 213 (8·2%) were pregnant women, 22 767 (6·4%) healthcare personnel, 96 626 (27%) were persons with chronic illness, and 207 296 (58%) were persons ≥50 years of age. A total of 2089 persons from the target populations were interviewed for the survey (Table1). Respondents were equally distributed across the four provinces; the median age was 47 years (range, 5–94 years) and more women than men participated. The majority (94%) of survey participants (excluding healthcare personnel) received vaccine in their village, followed by district and provincial hospitals. Median time between vaccination and survey interview was 7 days (range, 2–22 days). All interviews were completed within 3 weeks of vaccine administration.

**Table 1:** Demographics of survey respondents by target group, Lao PDR 2012

		Pregnant women (*n* = 187, 9%)	Persons ≥50 years of age (*n* = 819, 39%)	Persons with chronic disease (*n* = 733, 35%)	Healthcare personnel (*n* = 343, 16%)	Total (*n* = 2082)*
Sex	Females	187 (100%)	528 (64%)	544 (74%)	274 (80%)	1533 (74%)
	Males	–	291 (35%)	189 (26%)	69 (20%)	549 (26%)
Age in years	Median (range)	25 (15–45)	58 (50–94)	42 (5–86)	40 (19–65)	47 (5–94)
Province	Luang Prabang	62 (33%)	227 (28%)	170 (23%)	61 (18%)	520 (25%)
	Vientiane	16 (8·6%)	172 (21%)	187 (26%)	160 (47%)	535 (26%)
	Savanakhet	33 (18%)	227 (28%)	166 (23%)	63 (18%)	489 (23%)
	Champasak	76 (41%)	193 (24%)	210 (29%)	59 (17%)	538 (26%)

* Of the 2089 total interviewed respondents, seven people reported being in an “other” risk group and are not displayed in the table.

**Figure 1:**
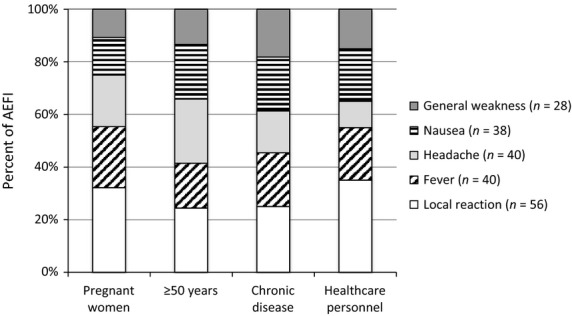
Frequency of adverse events following immunizations by risk group, by target group, Lao PDR 2012.

Two hundred and sixty-one (12·5%) survey participants self-reported a mild or moderate local reaction or systemic symptom (grade 1 or 2) (Table2, Figure1). The overall frequency by type was as follows: generalized weakness 28 (1·3%), nausea 38 (1·8%), headache 40 (1·9%), fever 40 (1·9%), and local reaction 56 (2·7%). No severe or life-threatening AEFI (grades 3 or 4) or hospitalizations were reported in this assessment. Among participants reporting an AEFI, the majority were persons with chronic diseases or persons ≥50 years of age (Table2). Among persons with an AEFI, the most common symptoms were local reactions and the frequency by risk group was similar (Figure1). Forty-three (16%) persons sought medical care due to their AEFI; 67% at a private clinic or pharmacy, a district hospital (21%), or a local health unit or provincial hospital (10%). Thirteen percent (33/261) indicated the AEFI led them to stop their daily activities for an average of 2·4 days (range, 1–7 days). Most pregnant women (56%) received vaccine in the second term of pregnancy (range, 1–8 months). Three gave birth after receiving vaccine; all had normal, full-term deliveries. Overall, 72% of survey participants heard about the vaccine via awareness activities prior to vaccination. The majority (61%) learned of the campaign from their village head, followed by healthcare workers or media sources (television or radio). Most would receive the vaccine annually (99%) and would like vaccine for their children (95%). The primary reason for vaccination was the health benefits provided to the recipient and their family. Thirty-one percent reported receiving the pandemic vaccine previously.

**Table 2:** Prevalence of adverse events within 7 days following seasonal influenza vaccine, *n* = 2089 vaccine recipients. Lao PDR 2012

	No. interviewed	% of all interviewed	AEFI	% of all AEFI (95% CI)
Total	2089		261	12·5%
Target group				
Pregnant women	187	9·0%	23	12·3% (7·9–16·7)
Persons aged ≥50 years	819	39%	73	8·9% (6·9–10·9)
Persons with chronic disease	733	35%	98	13·4% (10·2–16·6)
Healthcare personnel	343	16%	67	19·5% (12·1–26·9)
Others	7	0·3%	0	0%
Province				
Luang Prabang	520	25%	58	11·2% (9·7–12·5)
Vientiane capital	535	26%	94	17·6% (11·7–23·5)
Savannakhet	489	23%	40	8·2% (4·4–12·0)
Champasak	545	26%	69	12·7% (8·1–17·3)
Sex				
Female	1539	74%	210	13·6% (11·1–16·2)
Male	550	26%	51	9·3% (6·0–12·6)
Ethnic group				
Lao Loum	1646	79%	227	13·8%
Lao Theung	418	20%	33	7·9%
Lao Soung	16	0·8%	1	6·3%
Other	9	0·4%	0	0%

## Discussion

Following introduction of a seasonal influenza vaccine campaign in Lao PDR, the numbers of self-reported AEFI were low and mild in nature, as expected from this well-evaluated vaccine.^1^,^2^ Further, influenza vaccine was considered highly acceptable and nearly three-fourths of respondents had heard of influenza vaccine and the campaign prior to vaccination, indicating successful sensitization and social mobilization campaigns prior to the campaign.

The frequency of AEFI identified in the Lao population was similar to that seen in other studies of inactivated influenza vaccine.^11^,^8^ In placebo-controlled trials among adults, vaccine and placebo groups typically had similar rates of headache, myalgia, and malaise. A randomized controlled trial (RCT) of persons age 60 or older found a similar frequency of local reactions among the vaccine and placebo group (18%) and no difference in systemic adverse events.^12^ Our observational results were similar to the results from a RCT of healthy working adults; the vaccine group frequency of fever was 6·2%, headache 10·8%, and malaise 16%.^13^

This assessment was based on self-reported interview and was not confirmed by a clinician; therefore, objective classification of a mild, moderate, or severe event was difficult to ascertain. With a small sample size, we likely missed rare, severe AEFI. However, given the novelty of the vaccine campaign in this adult population, such events were likely to have been reported in the passive surveillance system. The majority of AEFIs were reported among persons ≥50 years of age or with chronic disease, and it is likely that some reported events were associated with an existing underlying medical condition and not the vaccine. The survey found daily activities were not affected among most participants which further substantiates that reported AEFIs were likely mild to moderate.

Survey responses indicated a high level of satisfaction among vaccine recipients, likely due to self-selection bias. Future assessments to better ascertain levels of interest and acceptance of vaccine should be made among the general population. Our results indicate the NIP campaign to generate awareness of the vaccine was effective in reaching a majority of the targeted population, findings from this study attest to the feasibility of introducing seasonal influenza vaccine in low- and middle-income countries.

The success of this vaccine campaign was in large measure a function of integration with existing NIP systems which are ideally suited to deliver vaccine to populations with limited access to preventive health services in a short period of time. The use of the NIP also contributed to building trust and acceptance of the vaccine among government stakeholders and the local population.

Following the successful introduction of seasonal influenza vaccine in 2012, Lao PDR has developed a plan to continue to provide seasonal influenza vaccine to priority populations, with a focus on pregnant women and continued to provide seasonal influenza vaccine to priority populations.^14^ Establishing feasibility, safety, and acceptability of the introduction of a new vaccine provides additional evidence for countries to commit to pursuing a disease reduction strategy through vaccination policy.

## Author contributions

Manilay Phengxay contributed to the design, execution, and analysis of the study and writing of the manuscript and final content. Sara Mirza contributed to the design, execution, and analysis of the study and writing of the manuscript and final content. Rita Reyburn contributed to the design, execution, and analysis of the study and reviewed final content. Anonh Xeuatvongsa contributed to the coordination, design, and execution of the study and reviewed final content. Christian Winter contributed to the design, execution, and writing of the manuscript and reviewed final content. Hannah Lewis contributed to the design execution and writing of the manuscript and reviewed final content. Sonja J. Olsen contributed to the analysis of the study and writing and editing of the manuscript and reviewed final content. Reiko Tsuyuoka contributed to the organization, design and execution of the study and reviewed final content. Viengphone Khanthamaly contributed to the design, execution, and writing of the manuscript and reviewed final content. Francisco S. Palomeque contributed to the analysis of data and reviewed final content. Joseph Bresee contributed to the organization, design, analysis, and writing of the manuscript and reviewed final content. Ann Moen contributed to the organization, design, analysis, and writing of the manuscript and reviewed final content and Andrew Corwin contributed to the organization, design, analysis, and writing of the manuscript and reviewed final content. The Lao PDR Field Epidemiology Training Cohort Team^3^ listed by name (Appendix 1), collected all data, entered all data, and analyzed data for this project as part of their training module. They all reviewed final content and contributed to the final product.

## Appendix

Bounlay Phommasack, Phengta Vongphrachanh, Bouaphanh Khamphaphongphane, Viengsavanh Kitthiphong, Khansay Sengsaya, Phonetavy Khodsimeuang, Phonephay Louangpaseuth, Khonesavanh Phanthavong, Phouthone Souliphone, Houmpheng Thionkeo, Khamphet Louanglad, Anousone Fongmany, Phoukhong Vorachansy, Boungnasith Khongsamphanh, Nouda Prasith, and Sisavath Phanatda.
